# Separation of realized ecological niche axes among sympatric tilefishes provides insight into potential drivers of co‐occurrence in the NW Atlantic

**DOI:** 10.1002/ece3.6745

**Published:** 2020-09-22

**Authors:** Jill A. Olin, Oliver N. Shipley, Robert M. Cerrato, Paul Nitschke, Cédric Magen, Michael G. Frisk

**Affiliations:** ^1^ Great Lakes Research Center Michigan Technological University Houghton Michigan USA; ^2^ School of Marine & Atmospheric Sciences Stony Brook University Stony Brook New York USA; ^3^ NOAA‐NMFS Northeast Fisheries Science Center Woods Hole Massachusetts USA; ^4^ Chesapeake Biological Laboratory University of Maryland Solomons Maryland USA

**Keywords:** *Caulolatilus microps*, continental shelf, habitat segregation, *Lopholatilus chamaeleonticeps*, resource use, spatial variability, stable isotope ratios

## Abstract

Golden and Blueline Tilefish (*Lopholatilus chamaeleonticeps* and *Caulolatilus microps*) are keystone taxa in northwest (NW) Atlantic continental shelf‐edge environments due to their biotic (trophic‐mediated) and abiotic (ecosystem engineering) functional roles combined with high‐value fisheries. Despite this importance, the ecological niche dynamics (i.e., those relating to trophic behavior and food‐web interactions) of these sympatric species are poorly understood, knowledge of which may be consequential for maintaining both ecosystem function and fishery sustainability. We used stable isotope ratios of carbon (δ^13^C) and nitrogen (δ^15^N) to build realized ecological niche hypervolumes to serve as proxies for diet and production use patterns of *L*. *chamaeleonticeps* and *C. microps*. We hypothesized that: (a) species exhibit ontogenetic shifts in diet and use of production sources; (b) species acquire energy from spatially distinct resource pools that reflect a sedentary life‐history and differential use of the continental shelf‐edge; and (c) species exhibit differentiation in one or more measured niche axes. We found evidence for ontogenetic shifts in diet (δ^15^N) but not production source (δ^13^C) in both species, suggesting a subtle expansion of measured ecological niche axes. Spatial interpolation of stable isotope ratios showed distinct latitudinal gradients; for example, individuals were ^13^C enriched in northern and ^15^N enriched in southern regions, supporting the assertion that tilefish species acquire energy from regional resource pools. High isotopic overlap was observed among species (≥82%); however, when hypervolumes included depth and region of capture, overlap among species substantially decreased to overlap estimates of 15%–77%. This suggests that spatial segregation could alleviate potential competition for resources among tilefish species inhabiting continental shelf‐edge environments. Importantly, our results question the consensus interpretation of isotopic overlap estimates as representative of direct competition among species for shared resources or habitats, instead identifying habitat segregation as a possible mechanism for coexistence of tilefish species in the NW Atlantic.

## Graphical abstract

We combine stable isotope ratios of carbon and nitrogen with environmental data to evaluate ecological niche dynamics of two ecologically and economically important deep‐water tilefish species. Through sampling individuals across much of their geographic range, we find evidence for limited movement of tilefish, as well as subtle niche expansion through ontogeny. Additionally, we find that tilefish use similar prey resource pools, but separate on fine‐scale depth preferences; a potential mechanism to alleviate interspecific competition in resource‐limited systems. Our analyses provide insight into the potential drivers of tilefish co‐occurrence, knowledge that may be consequential for maintaining both ecosystem function and fishery sustainability.

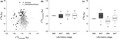

## 1 INTRODUCTION

The competitive exclusion principle predicts that under certain ecological conditions (e.g., resource limitation), coexisting species will differentiate aspects of their ecological niche to buffer competitive interactions or face extinction (Gause, 1934; Hardin, 1960; see also Levine & HilleRisLambers, 2009). This necessitates coexisting species to use unique regions of ecological niche space across environmental and biological axes, such as microhabitats (Brandl, Robbins, & Bellwood, 2015; Ebersole, 1985), temperature regimes (Wittman et al., 2010), peak activity patterns (Hayward & Slotow, 2009), and prey resources (Aguirre, Herrel, Van Damme, & Matthysen, 2002; Matich et al., 2017). The specific architecture of the ecological niche ultimately dictates the distributions of species in space and time, setting the geographic bounds of their functional role and broader influence across ecosystems (Rosado, Figueiredo, de Mattos, & Grelle, 2016).

Ecological niches are inherently complex, representing an infinite number of biotic and abiotic dimensions, across different levels of organization (Hutchinson, 1957). Subsequently, ecologists have investigated subsets of niche axes, largely based on concepts of Grinnell (1917) and Elton (1927). Grinnellian niches relate to abiotic processes such as temperature and salinity that influence the distribution of a species across broad scales. Eltonian niches, in contrast, relate to biotic processes such as resource‐use dynamics and predator–prey interactions that define a species role on its environment occurring at more regional scales (Cooper, Jetz, & Freckleton, 2010; Hutchinson, 1957; Rosado et al., 2016). These concepts form the basis for Hutchinson's definitions of fundamental and realized niches. Fundamental niches are defined by the theoretical range over which species could reside and reproduce, whereas realized niches reflect fundamental niches, in addition to, interspecies interactions. Further, from the perspective of ecological scale, the evaluation of regional species interactions is logistically more achievable than those across a species theoretical geographic range, that is, the fundamental niche (Bearhop, Adams, Waldron, Fuller, & MacLeod, 2004; Dézerald et al., 2018; Jackson, Inger, Parnell, & Bearhop, 2011; Petta et al., 2020). The complex interplay of biotic and abiotic processes could confound interpretation of realized ecological niches if only a single axis is quantified; for example, sympatric species may feed on similar prey or functional groups while partitioning habitat (Poulakis et al., 2017). The use of a single niche axis, therefore, could bias the attributed functional roles of species within ecosystems if the metrics provide markedly different results, but are assumed to represent the ecological niche (Shipley & Matich, 2020).

Studies exploring realized ecological niches have increased considerably in recent years, in part attributable to the evolving tools used to quantify various niche axes (Shipley & Matich, 2020; Thuo et al., 2019). The use of stable isotope ratios of carbon and nitrogen (δ^13^C and δ^15^N) to characterize aspects of an organisms' trophic ecology has revolutionized our understanding of ecological niches (Newsome, Martinez del Rio, Bearhop, & Phillips, 2007; Shipley & Matich, 2020) as these ecogeochemical tracers represent a spatio‐temporal integration of habitat and prey resource use in a consumer's tissues. Despite being commonly used to define species trophic behavior, recent evidence suggests that stable isotope ratios provide different information compared with more traditional approaches such as stomach content analyses, because the time periods over which the two techniques integrate information differs (Hette‐Tronquart, 2019; Petta et al., 2020). Therefore, understanding patterns of isotopic variability, and drivers therein, provides insight into complementary and divergent niche characteristics between co‐occurring species within a temporal window dictated by the isotopic incorporation rate of the analyzed tissues (Thomas & Crowther, 2015; Vander Zanden, Clayton, Moody, Solomon, & Weidel, 2015).

In food‐limited environments, such as deep‐water ecosystems, the necessity for species to differentiate ecological niche components may be greater than for those occupying highly productive systems (Demopoulos, McClain‐Counts, Ross, Brooke, & Mienis, 2017). In this case, resource limitation may drive increased interspecific competition, which may be alleviated by exploiting unique regions of niche space (e.g., exhibit distinct dietary or spatial resources; Carrassón & Cartes, 2002; Fock, Uiblein, Köster, & von Westernhagen, 2002; Preciado et al., 2017). Malacanthid species, specifically Golden (*Lopholatilus chamaeleonticeps*) and Blueline (*Caulolatilus microps*) Tilefish are integral components of the NW Atlantic Ocean shelf‐edge and slope environments where they maintain ecosystem structure and function through dual trophic and engineering functional roles (Coleman & Williams, 2002) and as such, have been labeled as a keystone species for supporting these ecosystems. Tilefishes in the NW Atlantic are assumed to display similar ecological niche characteristics; for example, species exhibit shifts in prey preference throughout ontogeny, as well as high dietary similarity (Ross, 1982; Steimle, Zetlin, Berrien, Johnson, & Change, 1999). Tilefishes also have patchy distributions with a propensity for high site fidelity that may be linked to thermal and sediment preference for burrow construction (Able, Grimes, Cooper, & Uzmann, 1982; Able, Twichell, Grimes, & Jones, 1987; Grimes, Able, & Jones, 1986; McBride, Vidal, & Cadrin, 2013; Nitschke & Miller, 2016). It is therefore possible, that current understanding of NW Atlantic tilefishes ecological niches fail to account for the potential intricacies occurring within and between species and similarity in aspects of their biology may increase interspecific interactions. Therefore, evaluating intra‐ and interspecific resource and habitat‐use dynamics could provide insight into the drivers of species coexistence.

In addition to their noted ecological importance, tilefishes support productive commercial and recreational fisheries in the NW Atlantic (Kitts, da Silva, & Roundtree, 2007). For example, commercial landings reported for *L. chamaeleonticeps* between 2007 and 2011 north of North Carolina were valued at $4.2–5.6 million annually (NOAA Fisheries, 2012). Although the fishery has existed since the 1900s, tilefishes are among some of the most data‐deficient of all targeted finfish species (Nitschke, 2017; SEDAR, 2017). Coupled with their *K*‐selected life‐history characteristics (i.e., slow growth, late maturation and long‐lived), high rates of fisheries exploitation have resulted in localized extirpations (McBride et al., 2013), such that the sustainability of NW Atlantic populations is a primary management concern (Nitschke, 2017; SEDAR, 2017). The limited observations and knowledge of the ecological roles of these species in shelf‐edge and slope environments restrict the capacity for ecosystem‐based management, which is pertinent given the increasing exploitation of these populations in recent years (Coleman & Williams, 2002; Trueman et al., 2014).

In this study, we combine carbon and nitrogen stable isotope data with biological (body size) and spatial (capture location, depth) measures to characterize the drivers of ecological niche variability in *L. chamaeleonticeps* and *C. microps*. We hypothesized that *L*. *chamaeleonticeps* and *C*. *microps* would (a) undergo enrichment of ^15^N and ^13^C with body size, indicative of ontogenetic shifts in diet and habitat use (Sánchez‐Hernández et al., 2019), (b) exhibit spatially distinct isotopic composition aligned with regional isotopic baselines (Oczkowski, Kreakie, McKinney, & Prezioso, 2016; Shipley, Olin, Power, Cerrato, & Frisk, 2019), and (c) exhibit differentiation in one or more of the measured niche axes. The coupling of isotopically derived ecological niche information with spatial and habitat characteristics offers insights into the potential drives of co‐occurrence among these understudied deep‐water species and provides a framework for future studies describing drivers of niche dynamics.

## 2 MATERIALS AND METHODS

### 2.1 Sample collection

Muscle tissue samples were collected from *L*. *chamaeleonticeps* and *C*. *microps* sampled from a depth range of 75–310 m across shelf waters of the NW Atlantic Ocean, from the south flank of Georges Bank to Cape Hatteras, during a fisheries‐independent survey using a stratified random design conducted in July and August 2017 (see Frisk, Olin, Cerrato, Nitschke, & Nolan, 2018). Individuals were captured using bottom long‐lines that consisted of a one‐nautical mile steel cable mainline equipped with 150 evenly spaced gangions baited with squid. Detailed survey methods are reported in Frisk et al. (2018). Tilefishes were measured for fork length (cm) weighed (g), sexed (via examination of gonads upon dissection in the field), and sampled for white muscle (∼2 g). Tissue samples were immediately frozen in the field and then transferred to a −20°C freezer in the laboratory. For each sampling event, regional location (e.g., Georges Bank, Southern New England, Mid‐Atlantic Bight) based on latitude and longitude, and depth (m) were recorded.

### 2.2 Stable isotope analysis

White muscle tissue samples were oven‐dried at 60°C for ≥72 hr and homogenized into a fine powder using a mortar and pestle. Ground muscle tissue was weighed into tin capsules (0.48–0.58 μg) and relative abundances of carbon (^13^C/^12^C) and nitrogen (^15^N/^14^N) were determined on a Thermo Finnigan Delta V Plus mass spectrometer (Thermo Finnigan) coupled with an elemental analyzer (Costech) at the University of Maryland's Center for Environmental Sciences, Chesapeake Biological Laboratory (Solomons, Maryland, USA). The results are expressed in standard delta notation (δ), defined as parts per thousand (‰) as follows: δ = [(*R*_sample_/*R*_standard_) − 1] × 10^3^, where *R* is the ratio of heavy to light isotope in the sample and standard, respectively (Coplen, 2011; Peterson & Fry, 1987). Machine error did not exceed 0.20‰ and 0.30‰, respectively, for δ^13^C and δ^15^N. The analytical precision based on the standard deviation of two internal standards (i.e., Acetanilide, Bass protein; run two every ten samples) was 0.15‰ and 0.04‰ for δ^13^C, and 0.12‰ and 0.11‰ for δ^15^N, respectively.

Lipids are mainly composed of carbon and are more depleted in ^13^C than protein and carbohydrates in fish tissues (Sweeting, Polunin, & Jennings, 2006). To adjust for this, we used the ratio of total organic carbon to total nitrogen (C:N) of untreated samples to mathematically correct δ^13^C values (δ^13^C_corr_) for any sample with a C:N ≥ 3.3 following a mathematical lipid normalization model derived from deep‐water fishes (Hoffman & Sutton, 2010).

### 2.3 Data analysis

All statistical analyses were performed in R (version 3.4.1, R Development Core Team, 2017) within the RStudio interface (version 1.0.136, R Studio Team, 2017). The level of significance (α) was set at 0.05. Prior to statistical analyses, we evaluated data for normality and homoscedasticity by visually examining probability plots and boxplots.

Differences in δ^13^C_corr_ and δ^15^N values among and within species were tested using analysis of variance (ANOVA), followed by Tukey's post hoc test, when significant differences were identified. Life‐history stage classifications were based on estimates of species‐specific maximum fork length (*L*_max_; Turner, Grimes, & Able, 1983; SEDAR, 2017) and fork length at maturity (*L*_mat_; McBride et al., 2013; Harris, Wyanski, & Mikell, 2004) for each species. The relationship between fork length and δ^13^C_corr_ and δ^15^N was estimated using linear regressions. Significant relationships would suggest ontogenetic shifts in primary production source (δ^13^C) and diet (δ^15^N).

To identify the geographical (region, depth) and/or biological (fork length) predictors of tilefish stable isotope values, we fitted generalized additive models (GAMs; Wood, 2006). GAMs utilize a smoothing function that can easily handle nonlinear relationships and uncover hidden structure between variables missed by traditional linear methods (Guisan, Edwards, & Hastie, 2002). GAMs were fitted assuming a Gaussian distribution for the stable isotope values and an identity link function to fit the response: δ ~ *s*(Depth) + *s*(Fork length) + Region, where *s* is the smoothing function applied to each continuous covariate. Each fish was assigned to a region (GB = Georges Bank, SNE = Southern New England, MAB = Mid‐Atlantic Bight) based on latitude and longitude of capture, and region was included as a categorical variable in the models. All GAMs were built with the package “mgcv” (Wood, 2006). All variables were tested for collinearity by evaluating their variance inflation factor (VIF), and since VIF values never exceeded 2, no collinearity was detected among the variables selected. All potential model configurations were given equal a priori weights, and the dredge function in the package “MuMIn” (Barton, 2019) was used to perform automated model selection to compare all potential model combinations. Model selection was based on the small sample size‐adjusted Akaike information criterion (AICc; Akaike, 1973; Burnham & Anderson, 2002). The top three models were compared with Akaike weights (*w_i_*) and evidence ratios (ER). The *w_i_* provides the probability of each model, given the data and full set of models and ER indicate how many times more likely the top model is compared to candidate models (Wagenmakers & Farrell, 2004).

Empirical Bayesian kriging (Pilz & Spöck, 2008) procedures in ArcGIS (version 10.4.3) were used to spatially interpolate stable isotope patterns generated for each species. The resulting spatial contour maps were used to delineate and define distinct isotopic regions based on the variation in isotopic values between sampling locations. We also include spatial interpolations of standard error, in line with previous studies (Ceriani et al., 2014), where higher error is associated with areas of high isotopic variability across individuals sampled at the same location, or low sampling effort. Bayesian kriging approaches generate multiple semi‐variograms that depict the spatial autocorrelation of the measured sample points and are used to interpolate and calculate error estimates for datasets with small sample sizes (Ceriani et al., 2014). Such approaches are useful to examine distinct spatial patterns occurring in any measured environmental and/or biological parameter and to infer spatial heterogeneity in isotopic composition (e.g., Hobson, Wunder, Van Wilgenburg, Clark, & Wassenaar, 2009; McMahon, Hamady, & Thorrold, 2013; Ceriani et al., 2014; Shipley et al., 2019). Stable isotope ratios are influenced by body size, and as such, contoured data (δ^13^C_corr_, δ^15^N) were standardized to species‐specific average fork length when body size‐specific relationships were observed. Multiple data points in the same location were averaged using the mean isotopic value.

To examine isotopic variation within (i.e., between life‐history stages) and between species, we calculated three isotopic metrics: δ^13^C_corr_ range (CR) and δ^15^N range (NR; Layman, Arrington, Montaña, & Post, 2007), and standard ellipse area (SEA; Jackson et al., 2011). CR is used to infer variability in production resources at the base of the food web, whereas NR is used to assess trophic diversity exhibited by individuals across the sampling community (Layman et al., 2007; Saporiti et al., 2016). SEA estimates were calculated with package “Stable Isotope Bayesian Ellipses in R” (SIBER; Jackson et al., 2011) using a maximum likelihood approach, and represent a bivariate estimation of isotopic niche width based on 40% of the data (Das et al., 2017; Jackson et al., 2011). Due to variable sample sizes for the sampled populations, we calculated small sample size‐corrected (SEA_C_) and Bayesian estimates of SEA (SEA_B_ [median values]). Bayesian estimates are based on 10,000 posterior draws and were trimmed by the first 1,000 to account for the model burn‐in period and improve error estimation (Jackson et al., 2011). Ecological niche overlap within and between species was determined using methods outlined in the R package “nicheROVER” (Swanson et al., 2015). We used a Bayesian approach to calculate the intraspecific (i.e., among life‐history stages of *L. chamaeleonticeps*) and interspecific (i.e., between mature life‐history stages of the two species) probability that an individual from species A is found in the isotopic niche area of species B, and vice versa, using (a) isotopic indicators of niche (e.g., bivariate stable isotope data), and (b) a combination of isotopic indicators with geographical indicators of niche (e.g., region and depth) (Swanson et al., 2015). We used ellipses incorporating 95% of data to calculate isotopic overlap (overlap estimates run for 10,000 iterations) to balance type I and type II errors and account for individual variability across the sampled population (Shipley et al., 2019).

## 3 RESULTS

### 3.1 Isotope values and interactions

**FIGURE 1:**
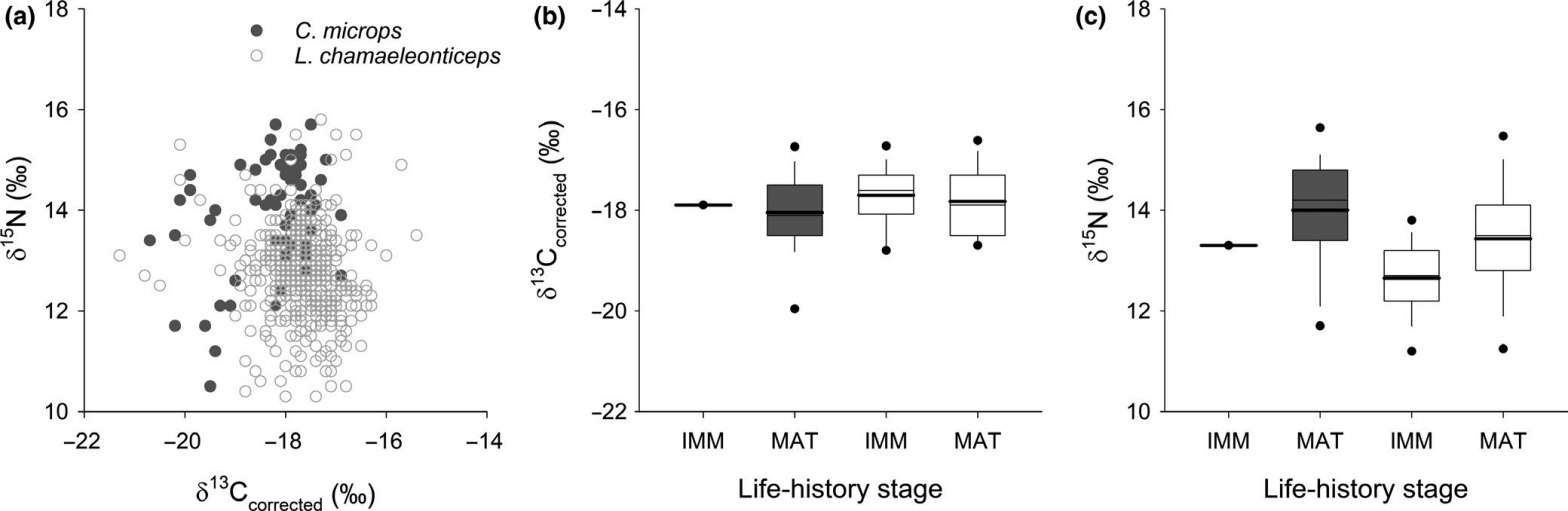
Isotope biplot (a) and boxplots of δ^13^C_corr_ (b) and δ^15^N (C) values derived from immature (IMM) and mature (MAT) life‐history stages of *Caulolatilus microps* (gray) and *Lopholatilus chamaeleonticeps* (white) from the NW Atlantic. Data are mean (thick line), median (thin line), 25th and 75th percentiles (box), and the 5th and 95th percentiles (points)

**FIGURE 2:**
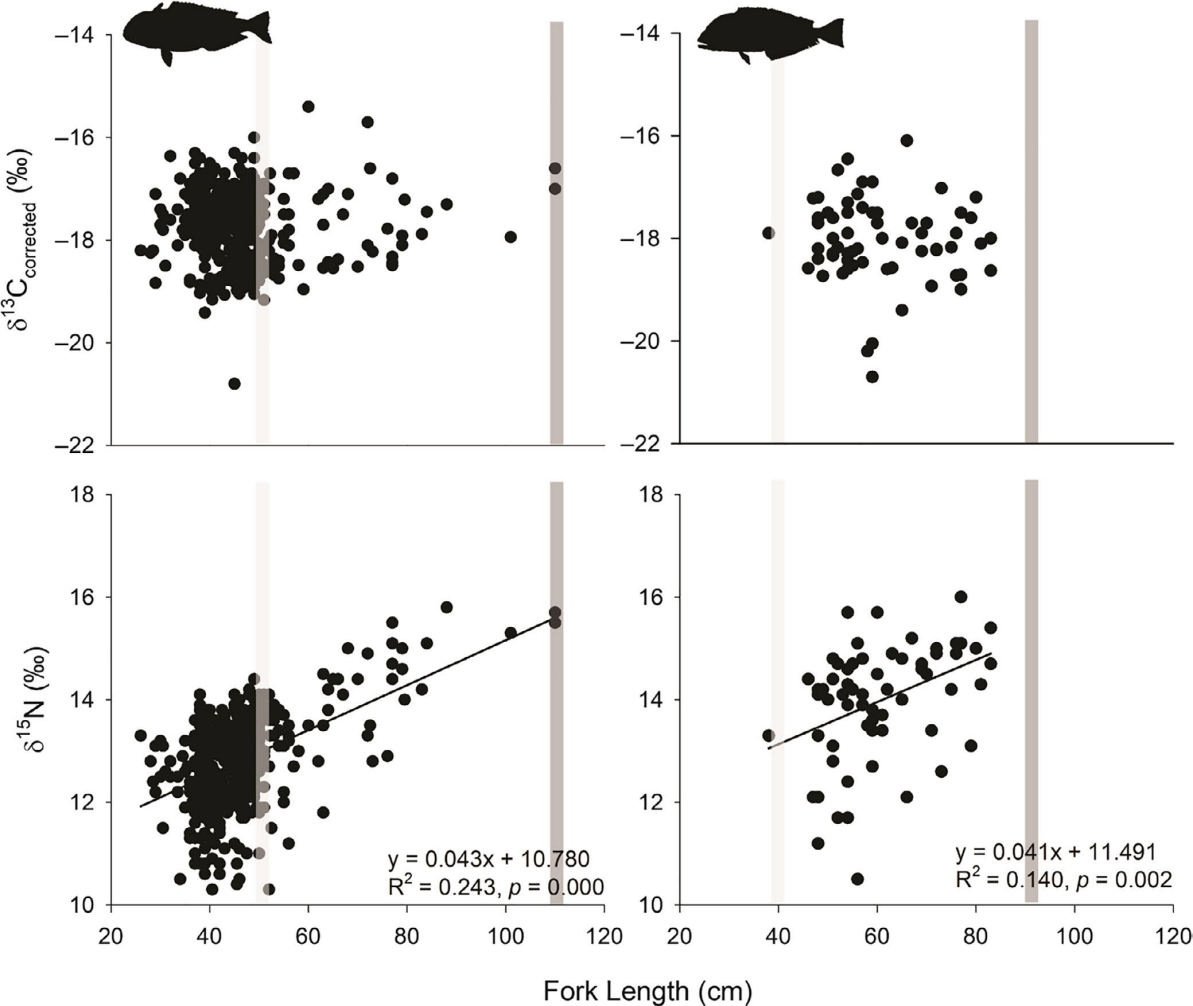
Linear regressions illustrating body size (fork length) relationships of δ^13^C_corr_ and δ^15^N values generated from *Lopholatilus chamaeleonticeps* (left panel) and *Caulolatilus microps* (right panel) of NW Atlantic. Species‐specific maximum fork length (*L*_max_; Turner et al., 1983; SEDAR, 2017) and fork length at maturity (*L*_mat_; McBride et al., 2013; Harris et al., 2004) are represented by the dark gray and light gray bars, respectively

Bulk δ^13^C_corr_ ranged from −20.8‰ to −15.4‰ and bulk δ^15^N ranged from 8.8‰ to 16.0‰ across species (Table 1, Figure 1a). Mean δ^13^C_corr_ of *L*. *chamaeleonticeps* (mean ± *SD*; −17.7 ± 0.7‰) was significantly higher (δ^13^C_corr_: *F*_1,547_ = 13.10, *p* < .001) than *C*. *microps* (−18.1 ± 0.8‰; Table 1). In contrast, mean δ^15^N of *L. chamaeleonticeps* (12.8 ± 0.9‰) was significantly lower (δ^15^N: *F*_1,547_ = 101.14, *p* < .001) than *C*. *microps* (14.0 ± 1.1‰; Table 1). Among mature life‐history stages, mean δ^13^C_corr_ did not differ (δ^13^C_corr_: *F*_1,547_ = 4.23, *p* = .056; Figure 1b) among species, but mean δ^15^N was significantly higher in *C*. *microps* (δ^15^N: *F*_1,547_ = 52.50, *p* < .001; Figure 1c) relative to *L*. *chamaeleonticeps*. Similarly, we observed no differences in mean δ^13^C_corr_ among immature and mature life‐history stages of *L*. *chamaeleonticeps*, but mean δ^15^N differed significantly (Figure 1c). Due to low sample size of immature *C*. *microps,* comparisons among life‐history stages for this species were not conducted. Linear regression analysis showed significant relationships between fork length and δ^15^N for both species (Figure 2). Significant relationships were not observed between fork length and δ^13^C_corr_ (Figure 2).

**TABLE 1:** Fork length (cm), depth range (m), stable isotope values (‰), and elemental ratios of muscle tissues from NW Atlantic tilefish by life‐history stage (IMM—immature, MAT—mature)

Species	Stage	*n*	Fork length	Depth range	δ^13^C_corr_	δ^15^N	C:N
*Lopholatilus chamaeleonticeps*	IMM	368	42.1 ± 4.6 (26.0–49.5)	136.2 ± 32.0 (93.3–‐288.9)	−17.7 ± 0.6 (−20.8 to −16.0)	12.6 ± 0.8 (8.8–14.4)	3.2 ± 0.3 (2.6–6.2)
	MAT	118	56.6 ± 13.1 (45.5–110.0)	144.8 ± 44.5 (95.1–292.6)	−17.8 ± 0.7 (−19.2 to −15.4)	13.2 ± 1.1 (9.9–15.8)	3.5 ± 0.5 (2.9–5.7)
*Caulolatilus microps*	IMM	1	38.0	104.2	−17.9	13.3	3.2
	MAT	64	61.1 ± 10.6 (46.0–83.0)	100.3 ± 17.1 (75.9–133.7)	−18.0 ± 0.8 (−20.7 to −16.1)	14.0 ± 1.1 (10.5–16.0)	3.9 ± 1.2 (3.1–8.4)

Note

Data are mean ± 1 *SD* and range.

### 3.2 Generalized additive models

The best models based on AICc describing the distribution of tilefish δ^13^C_corr_ and δ^15^N in the NW Atlantic accounted for 5.0%–41.7% of the explained deviance, with *C*. *microps* models garnering the highest explained deviance overall (Table 2). No clear best model was identified for δ^13^C_corr_ of *L*. *chamaeleonticeps*, as models that included region and depth exhibited ER of 1.3 (Table 2). For δ^15^N, the best model included fork length, depth, and region (Table 2), with response plots indicating an increase with fork length > 35 cm and depth > 250 m (Figure S1). In contrast, depth was the most influential variable on δ^13^C_corr_ (Table 2), with the best model for δ^13^C_corr_ being three times more likely than the other models (Table 2). The models for δ^15^N were not well differentiated for *C*. *microps* but suggest depth, fork length and region are important variables explaining δ^15^N (Table 2). Response plots showed consistent δ^13^C_corr_ between 80–120 m and higher δ^15^N with depths < 100 m and with body size (Figure S2).

**TABLE 2:** Summary of model selection using Akaike's information criterion (AIC_C_), log‐likelihood (logLik), weight (w*_i_*, the ratio of ∆AIC_C_ values for each model relative to the set of candidate models), the deviance explained [DE (%)], and evidence ratios (ER) for the stable isotope models for each tilefish species in the NW Atlantic Ocean

Model	*n*	Intercept ± *SE*	*df*	logLik	AIC_C_	w*_i_*	DE (%)	ER
*Lopholatilus chamaeleonticeps*								
δ^13^C_corr_ ~ *s*(Fork Length)	486	−17.7 ± 0.1	7	−470.52	955.62	0.33	5.0	1.0
δ^13^C_corr_ ~ *s*(Fork Length) + Region		−17.7 ± 0.1	8	−468.97	956.20	0.25	4.5	1.3
δ^13^C_corr_ ~ *s*(Fork Length) + *s*(Depth)		−17.7 ± 0.1	9	−468.92	956.22	0.24	4.5	1.4
δ^15^N ~ *s*(Fork Length) + *s*(Depth) + Region	486	12.9 ± 0.1	12	−553.82	1,133.86	0.99	30.9	1.0
δ^15^N ~ *s*(Fork Length) + Region		12.9 ± 0.1	8	−563.28	1,143.69	0.01	28.2	99.0
δ^15^N ~ *s*(Fork Length) + *s*(Depth)		12.7 ± 0.1	12	−564.57	1,154.46	0.00	27.8	99.0
*Caulolatilus microps*								
δ^13^C_corr_ ~ *s*(Depth)	64	−18.0 ± 0.1	7	−60.68	137.74	0.56	41.7	1.0
δ^13^C_corr_ ~ *s*(Depth) + Region		−18.9 ± 0.8	10	−57.70	139.87	0.19	46.0	2.9
δ^13^C_corr_ ~ *s*(Depth) + *s*(Fork Length)		−18.0 ± 0.1	7	−60.82	139.93	0.19	40.4	2.9
δ^15^N ~ *s*(Depth) + *s*(Fork Length)	64	14.0 ± 0.1	6	−85.34	184.50	0.59	34.5	1.0
δ^15^N ~ *s*(Depth) + *s*(Fork Length) + Region		15.8 ± 1.0	8	−83.29	185.32	0.39	38.3	1.5
δ^15^N ~ *s*(Depth)		14.0 ± 0.1	7	−88.10	192.79	0.01	28.2	59.0

Note

Best model resulted from lowest AIC_C_ and logLik estimates.

### 3.3 Geographic variability

**FIGURE 3:**
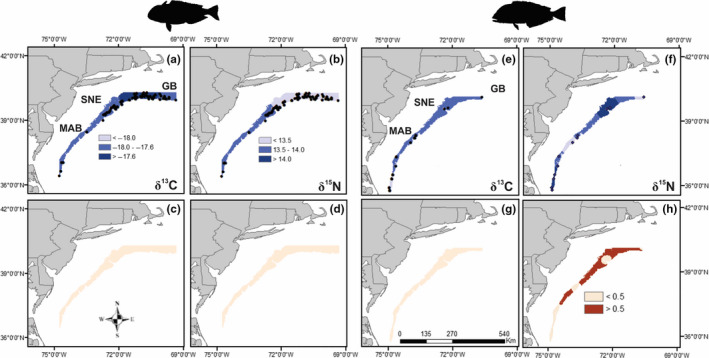
Spatial isoscapes generated from δ^13^C_corr_ (left panels) and δ^15^N (right panels) ratios of *Lopholatilus chamaeleonticeps* (a, b) and *Caulolatilus microps* (e, f)) with standard error (bottom panel) sampled regionally from the NW Atlantic Ocean. Areas with higher standard error likely reflect regions with high isotopic variability across individuals and/or lower sampling effort. Data are standardized by body size (fork length)

Empirical Bayesian kriging illustrated distinct spatial patterns in stable isotope ratios of both species. For example, a south to north gradient was observed in δ^13^C_corr_, with fishes sampled in the northern extent of the study area, specifically in SNE region, showing the highest values (Figure 3a,e). On average, δ^13^C_corr_ of *C*. *microps* was 0.2‰ lower than *L*. *chamaeleonticeps* across the sampled area. In contrast, higher δ^15^N was observed in fishes sampled in the southern extent of the sampling area, mainly in the MAB region (Figure 3b,f). On average, standardized δ^15^N of *L*. *chamaeleonticeps* was 1.5‰ higher than *C*. *microps* across the study area. Standard error values of interpolated data points were lower for *L*. *chamaeleonticeps* (Figure 3c,d) relative to *C*. *microps* (Figure 3g,h) but did not exceed 0.5 and 0.7, respectively.

### 3.4 Isotopic variability and overlap estimates

**FIGURE 4:**
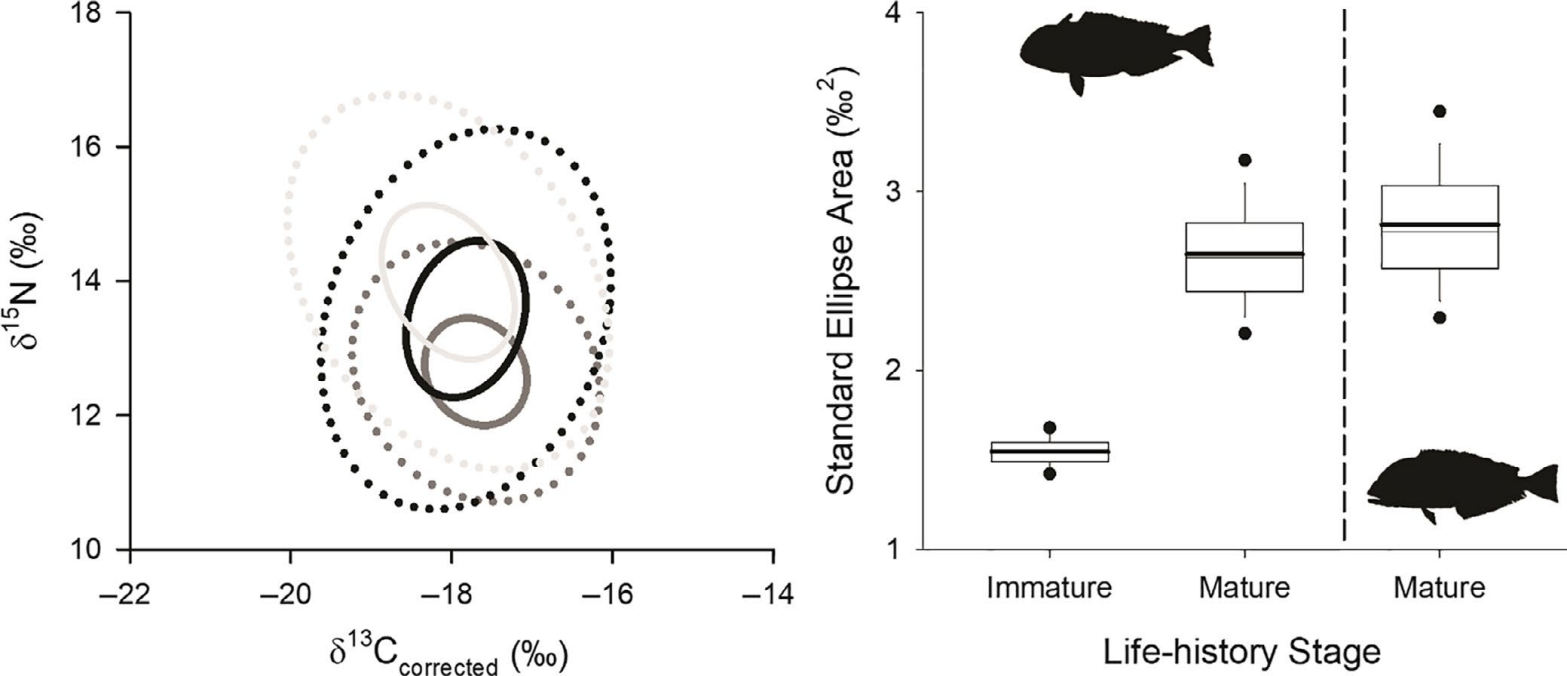
Left panel: Isotopic overlap (95% of data [used for niche overlap calculations], solid lines) and core isotopic niche (40% of data [SEA], dotted lines) estimates for both life‐history stages (immature—dark gray, mature—black) of *Lopholatilus chamaeleonticeps* and mature *Caulolatilus microps* (light gray). Right panel: SEA_B_ estimates with 25%, 50%, 75%, and 95% credible intervals (dark line represents maximum likelihood estimated SEA)

CR was greatest for immature *L*. *chamaeleonticeps* with mature *L*. *chamaeleonticeps* exhibiting the lowest estimates (Table 3). NR was greatest for mature *L*. *chamaeleonticeps* followed by immature *L*. *chamaeleonticeps* (Table 3). Similarly, SEA was greatest for mature relative to immature life‐history stages of *L*. *chamaeleonticeps*. Among species, SEA was greatest for mature *C*. *microps* compared with mature *L*. *chamaeleonticeps* (Table 3; Figure 4). These results were consistent with SEA_C_ and SEA_B_ estimates (Table 4; Figure 4). Considerable total isotopic overlap was observed among life‐history stages of *L*. *chamaeleonticeps* (>78.6%) and between mature individuals of both species (>82.9%; Table 4; Figure 4). Isotopic overlap estimates for life‐history stages of *L*. *chamaeleonticeps* changed minimally with addition of geographic covariates (Table 4). In contrast, isotopic overlap estimates for mature tilefish were influenced by geographic covariates. Most notably, overlap among species was greatly reduced when depth (38.0%) and region (15.8%) were incorporated into the analysis for *L*. *chamaeleonticeps* and *C*. *microps*, respectively (Table 4).

**TABLE 3:** Isotope niche metrics including δ^13^C_corr_ (CR) and δ^15^N (NR) ranges (‰), total area (TA), standard ellipse area (SEA), small sample size‐corrected SEA (SEA_C_), and Bayesian estimates of SEA (SEA_B_ median values]) for different life‐history stages of *Lopholatilus chamaeleonticeps* and mature life‐history stages of *Caulolatilus microps* from the NW Atlantic

Stage	*n*	CR	NR	TA	SEA (‰^2^)	SEA_C_ (‰^2^)	SEA_B_ (‰^2^)
*Lopholatilus chamaeleonticeps*							
Immature	368	4.8	5.6	14.8	1.5	1.5	1.5
Mature	118	3.8	5.9	14.2	2.6	2.7	2.6
*Caulolatilus microps*							
Mature	63	4.6	5.5	13.0	2.8	2.8	2.8

**TABLE 4:** Isotopic overlap (%) estimates between life‐history stages of *Lopholatilus chamaeleonticeps* and between mature life‐history stages of both species

	Stage B	Traditional		+ Depth		+ Region	
		Immature	Mature	Immature	Mature	Immature	Mature
Stage A	Immature	–	96.0	–	96.6	–	96.3
	Mature	78.6	–	73.6	–	76.4	–
	Species B	*L*. *chamaeleonticeps*	*C*. *microps*	*L*. *chamaeleonticeps*	*C*. *microps*	*L*. *chamaeleonticeps*	*C*. *microps*
Species A	*L*. *chamaeleonticeps*	–	82.9	–	76.7	–	59.2
	*C*. *microps*	90.6	–	38.0	–	15.8	–

Note

Overlap estimates represent comparisons of isotopic overlap of stage/species A compared to stage/species B, or vice versa (traditional). Covariates of depth and region are then added to the hypervolumes, and new overlaps are calculated.

## 4 DISCUSSION

The results of our study provide evidence for differentiation of ecological niche axes among sympatric tilefish species that occupy the continental shelf‐edge of the NW Atlantic Ocean. Specifically, the dietary and habitat‐use patterns of *L*. *chamaeleonticeps* were best described by body size and those for *C. microps* were strongly influenced by depth. Isoscapes generated for both species revealed distinct latitudinal gradients in δ^13^C and δ^15^N supporting the assertion that tilefishes acquire energy from regional resource pools that reflect their sedentary life histories. High levels of isotopic overlap among species that result from shared local and regional resource pools were indicated with isotopic analyses in isolation. However, the incorporation of multiple niche axes resulted in a notable reduction in isotopic overlap. This suggests fine‐scale spatial differentiation among species may be a mechanism to allow for shared use of regional resource pools. Importantly, this result questions the consensus interpretation of isotopic overlap estimates as representative of direct competition among species for shared resources or habitats, instead identifying habitat segregation as a potential mechanism for tilefish species coexistence in the NW Atlantic.

Studies evaluating *L*. *chamaeleonticeps* and *C*. *microps* diet suggest individuals are largely opportunistic, feeding on benthic‐associated prey, typified by crustaceans, annelids, mollusks, echinoderms, and fishes (Dooley, 1978; Freeman & Turner, 1977; Ross, 1982; Steimle et al., 1999), with a gradual shift in diet composition through ontogeny. For example, a greater proportion of echinoderms and mollusks was noted in stomach contents of juveniles, with increased incorporation of larger prey, such as fishes and decapods, as individuals grew and matured (Freeman & Turner, 1977; Ross, 1982). Our data imply a gradual expansion of diet, rather than directed shift from one major prey group to another, as body size explained < 25% of the total variance in δ^15^N across both species. The absence of direct diet‐switching is strengthened by the significant isotopic overlap occurring between immature and mature life‐history stages of *L*. *chamaeleonticeps* (i.e., >73%). Depth was also a significant predictor of tilefish δ^15^N with distinct differences among species, suggesting that ontogeny is not the only consideration for shifting δ^15^N values. Specifically, δ^15^N was higher in *L*. *chamaeleonticeps* at depths > 250 m compared to higher δ^15^N in *C*. *microps* at depths < 100 m. It is important to note, however, that few immature *C*. *microps* and no larval or young‐of‐year individuals of either species were captured in the present study, which limits definitive assertions regarding ontogenetic diet variability in these species to the body size classes sampled here.

Ontogenetic shifts in δ^13^C were weakly supported for the tilefish species and pairwise comparisons of δ^13^C between immature and mature life‐history stages of *L*. *chamaeleonticeps* were not significant. Though body size was identified as the most influential factor on *L*. *chamaeleonticeps* δ^13^C, the model performed poorly with only 5% of the deviance explained. This suggests that other factors (e.g., differences in isotopic routing and fractionation of ^13^C at the individual‐level or use of different production sources) may drive the observed variability in δ^13^C of *L*. *chamaeleonticeps* or that the observation and/or processing error is too high to detect significant variables. Depth was a significant predictor of δ^13^C in *C*. *microps*, with individuals captured from shallow depths exhibiting lower δ^13^C (i.e., ^13^C‐depleted). This implies that, if differential use of habitat across ontogeny does occur, it is subtle and in the case of *C*. *microps* may be more influenced by depth rather than body size. Although, this finding is consistent with catch data for *L*. *chamaeleonticeps* and *C*. *microps* from bottom trawl surveys conducted through NOAA's National Marine Fisheries Service in the NW Atlantic that show simultaneous capture of immature and mature life‐history stages (Nitschke & Miller, 2016; Steimle et al., 1999), the single immature *C*. *microps* included in this study does limit the strength of this conclusion. Assuming pelagic phytoplankton is the dominating primary production source, the stable isotope composition of sinking organic matter changes with depth due to preferential assimilation of the light isotopes during microbial metabolism (Lin et al., 2014; Mintenbeck, Jacob, Knust, Arntz, & Brey, 2007). This results in a general enrichment in ^13^C with increasing depth (Parzanini, Parrish, Hamel, & Mercier, 2017). The lack of relationship between depth and δ^13^C in *L*. *chamaeleonticeps* could be attributable to the depth range of individuals sampled compared with *C*. *microps,* as no *L*. *chamaeleonticeps* were sampled < 80 m; depths associated with the greatest ^13^C‐depleted values. Likewise, this could indicate greater cross‐slope mobility of *C. microps* throughout this depth range (e.g., vertical migration), as adults are capable of using a range of bottom types and reliefs (e.g., high relief, rocky outcroppings, gently sloping area; Ross & Huntsman, 1982; SEDAR, 2017).

Considering that closer association with benthic, as opposed to pelagic environments results in ^13^C enrichment (France, 1995) in an organism's tissues, ontogenetic shifts in δ^13^C would likely have been evident from a broader range of body sizes that included early life‐history stages (i.e., larval and young‐of‐year; Mittelbach & Persson, 1998; Galván, Sweeting, & Reid, 2010; Olin, Rush, MacNeil, & Fisk, 2012). Directed evaluation of movement in these species across their life history is largely absent from the literature. Current knowledge regarding site fidelity is based on simple observation (Able et al., 1982; 1987) and a single mark–recapture study of adults conducted in the Hudson Canyon implying minimal movement of *L*. *chamaeleonticeps* from established burrows (Grimes et al., 1986). Regardless, a greater understanding of movement patterns across life‐history stages, including larval and young‐of‐year individuals, should therefore be a future priority for resource managers particularly over smaller spatial scales given these species' patchy distributions.

Spatial isoscapes generated for *L*. *chamaeleonticeps* and *C*. *microps* highlighted distinct regional patterns in isotopic composition that generally align with regional phytoplankton, POM and DOM baselines across the NW Atlantic shelf‐edge and slope (Bauer, Druffel, Wolgast, & Griffin, 2001; Magozzi et al., 2017; McKinney, Oczkowski, Prezioso, & Hyde, 2010; Oczkowski et al., 2016). Specifically, a south to north latitudinal gradient was observed in δ^13^C and δ^15^N, with fishes sampled in northern latitudes exhibiting ^13^C enrichment and fishes sampled in southern latitudes showing ^15^N enrichment. These regional patterns are consistent with our initial hypothesis that *L*. *chamaeleonticeps* and *C*. *microps* would acquire energy from spatially distinct resource pools that reflect their sedentary life histories and thus differential exposure to regional isotopic baselines. Food webs associated with the continental shelf and slope habitats of the NW Atlantic are predominantly supported by pelagic phytoplankton‐based primary production (POM) although fluvial production from land run‐off may provide additional allochthonous sources of production to proximate regions from the coastline (Demopoulos et al., 2017; Oczkowski et al., 2016; Parzanini et al., 2017) that could explain the regional differences observed here. *Lopholatilus* *chamaeleonticeps* isotope values were more regionally distinct compared with *C*. *microps*. This may be due to sample size across the ranges sampled, as only 64 *C*. *microps* were analyzed compared with 486 *L*. *chamaeleonticeps*. Further, *C*. *microps* were predominantly captured south of the Hudson Canyon in the MAB region, whereas *L*. *chamaeleonticeps* catch was broadly distributed across the region. This was reflected in the standard error with higher error apparent in areas with limited samples.

The range of δ^13^C and δ^15^N observed for *C. microps* could reflect longer‐term movement patterns or preference for resource use across a more limited latitudinal range. Limited isotopic ranges were observed by Shipley et al. (2019) for Winter Skate *Leucoraja ocellate* across the NW Atlantic, and the authors attributed those findings to foraging in specified geographic areas, requiring extensive movements. These findings were later supported by passive acoustic telemetry (Frisk et al., 2019). Movement to specific foraging grounds seems unlikely for both *L*. *chamaeleonticeps* and *C*. *microps* given high site fidelity (Able et al., 1982; Grimes et al., 1986; Ross, 1982), patchy distributions in the NW Atlantic and propensity for specific habitat characteristics (Frisk et al., 2018). Extensive movements cannot be discounted entirely, and in fact, two studies have speculated the potential for seasonal habitat use of Georges Bank in winter months by *L*. *chamaeleonticeps* due to thermal preferences of the species (Freeman & Turner, 1977; Grimes et al., 1986). Continued research is needed to determine definitively if these species forage within localized or between spatially disparate food webs, but our results suggest the former. Defining the specific energy sources underpinning deep‐sea communities, as well as examining clear predator–prey relationships across species should be a focus of future work. Visual evaluation and DNA sequencing techniques of stomach contents, and fatty acid analyses may also provide more detail on the specific dietary preferences of each species (Olin et al., 2017; Poulakis et al., 2017). Regardless, the consistent isotopic gradients suggest that individuals of these two species may exhibit limited dispersal from the initial capture origin, and thus reflect the isotopic composition of regional resource pools. Such observations are in line with existing data on tilefishes more broadly, suggesting that individuals generally exhibit high site fidelity with limited dispersal as adults (Able et al., 1982; 1987; Ross & Huntsman, 1982; SEDAR, 2017), and presumably forage primarily within localized food webs.

In line with competitive exclusion principle, we predicted that the sympatric nature of *L*. *chamaeleonticeps* and *C*. *microps* would result in minimal overlap in one of the measured niche axes. Although we were unable to specifically categorize resource availability in this system, we describe the ecological niche characteristics that may promote species coexistence. Niche overlap estimates based solely on δ^13^C and δ^15^N do not support this hypothesis. Rather *L*. *chamaeleonticeps* and *C*. *microps* exhibited similarly sized (i.e., SEA estimates) and highly overlapping isotopic niches (>82.9%). This observation is consistent with stomach content studies that suggest the two species consume similar benthic‐associated prey (Dooley, 1978; Freeman & Turner, 1977; Ross, 1982; Steimle et al., 1999), possibly indicating that prey resources of *L*. *chamaeleonticeps* and *C*. *microps* may not be limited, allowing both species to target similar prey resources or that competitive interactions for prey could be high. However, the inclusion of additional niche axes, specifically depth, revealed niche differentiation among these sympatric species. Our findings are consistent with others, suggesting high dietary overlap could be mitigated by predators consuming the same prey but across different depth ranges (Preciado et al., 2017). A preference for depth could be achieved by moving a short distance along the shelf slope as compared to the flatter topography of the shelf. Thus, localized spatial segregation of the two species could result in fine‐scale segregation while the regional prey field remains relatively constant. More precise dietary and environmental information is needed to determine if the diets of these species change across their distribution or if the changes in isotope values observed in this study were indicative of changes in isotopic baselines. Regardless, in the absence of habitat‐specific preferences of these species, specifically depth and region of capture, could result in misleading conclusions regarding species interactions.

The above finding raises an important consideration, not just for the ecological interactions of tilefishes, but regarding the broader efficacy of stable isotope ratios to investigate competitive interactions between sympatric species. Our data suggest that, contrary to the conclusions of many published studies, isotopic niche overlap does not necessarily indicate direct competition for resources (Hette‐Tronquart, 2019). Thus, the inclusion of additional niche axes may be required to fully appreciate the degree of competitive interactions occurring between species; these data are often unreported in scientific studies (Shipley & Matich, 2020). A clear acknowledgment of the limitations of stable isotope ratios is an important component of ecological inference (Hette‐Tronquart, 2019), and our results suggest that studies should aim to employ a multitude of data types when building niche hypervolumes, particularly across broad resource axes (Petta et al., 2020; Shipley & Matich, 2020). The use of isotope ratios in isolation could confound the interpretation of species interactions and mechanisms that define their fundamental niche.

Our data provide new insights into the trophic ecology of NW Atlantic tilefishes and augment current understanding derived from stomach contents' studies. Results implied that body size‐ and depth‐specific patterns of resource use by tilefishes are mechanisms that may help support the coexistence of these species. This confirmation has important implications for reaching informed management decisions, such as guiding contemporary marine reserve designs and predicting the impacts of anthropogenic and natural stressors on these species' distributions (Drake, Randin, & Guisan, 2006). Whether further intricacies in resource‐use dynamics occur within and between tilefish populations remains unclear; however, examining a broader suite of environmental and biological niche axes is imperative to understanding the competitive interactions and long‐term persistence of these populations.

## CONFLICT OF INTEREST

The authors declare that the research was conducted in the absence of any commercial or financial relationships that could be construed as a potential conflict of interest.

## AUTHOR CONTRIBUTIONS

**Jill A. Olin:** Conceptualization (lead); data curation (lead); formal analysis (lead); funding acquisition (equal); investigation (lead); methodology (lead); writing – original draft (lead); writing – review & editing (lead). **Oliver N. Shipley:** Conceptualization (supporting); formal analysis (supporting); writing – original draft (supporting); writing – review & editing (supporting). **Robert M. Cerrato:** Conceptualization (supporting); formal analysis (supporting); funding acquisition (equal); writing – original draft (supporting); writing – review & editing (supporting). **Paul Nitschke:** Conceptualization (supporting); data curation (supporting); funding acquisition (supporting); writing – original draft (supporting); writing – review & editing (supporting). **Cédric Magen:** Data curation (supporting); writing – review & editing (supporting). **Michael G. Frisk:** Conceptualization (supporting); formal analysis (supporting); funding acquisition (lead); writing – original draft (supporting); writing – review & editing (supporting).

## Supporting information

Fig S1‐S2

## Data Availability

Data are available from the Dryad Digital Repository: https://doi.org/10.5061/dryad.qnk98sfcs (Olin et al. 2020).
