# Supplementary material for: Separation of realized ecological niche axes among sympatric tilefishes provides insight into potential drivers of co‐occurrence in the NW Atlantic
Source: Ecol Evol. 2020 Sep 22;10(19):10886–98. doi: 10.1002/ece3.6745 (PMC7548204; doi:10.1002/ece3.6745)
Supplement: Supplementary file 1 — Fig S1‐S2 [file ECE3-10-10886-s001.docx]

**SUPPLEMENTAL**

**Figure S1.** Partial GAM plots identifying the additive effect of geographical and biological variables on the distribution of stable isotope values of *Lopholatilus chamaeleonticeps* throughout the NW Atlantic.


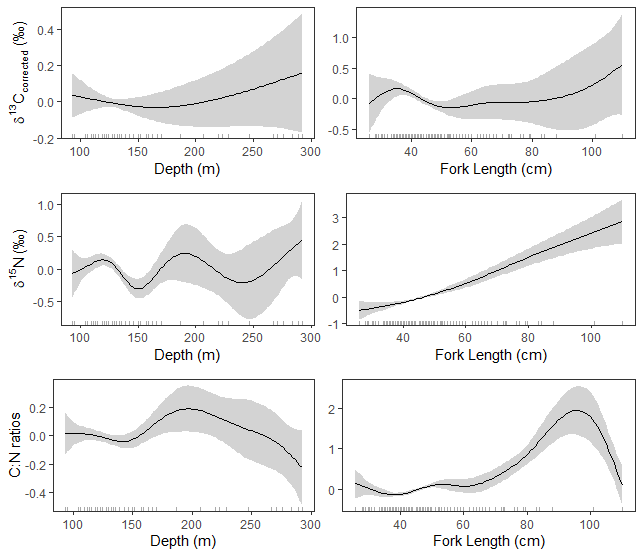


**Figure S2.** Partial GAM plots identifying the additive effect of geographical and biological variables on the distribution of stable isotope values of *Caulolatilus microps* throughout the NW Atlantic.

**
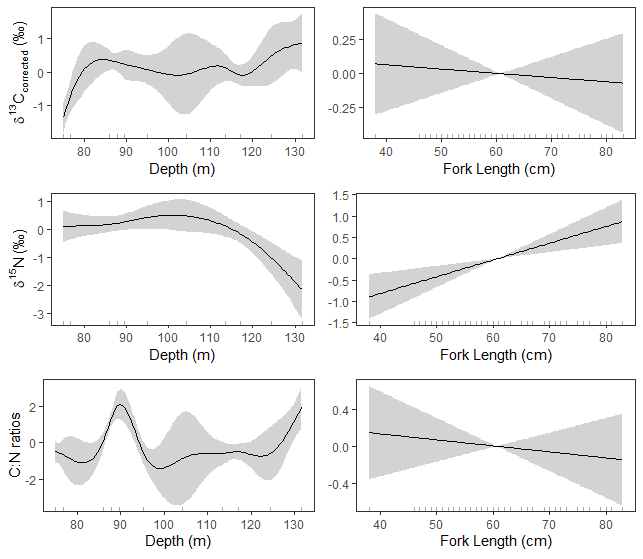
**
